# Curcumin induced oxidative stress causes autophagy and apoptosis in bovine leucocytes transformed by *Theileria annulata*

**DOI:** 10.1038/s41420-019-0180-8

**Published:** 2019-06-04

**Authors:** Prasanna Babu Araveti, Anand Srivastava

**Affiliations:** National Institute of Animal Biotechnology (NIAB), Hyderabad, India

**Keywords:** Antiparasitic agents, Parasitic infection

## Abstract

Bovine tropical theileriosis is a tick-borne disease, caused by *Theileria annulata* which is a protozoan parasite that resides within the B-cells and macrophages. *T. annulata* is a unique parasite that can transform bovine leucocytes which leads to the cancer hallmarks in the infected cells. Previously, curcumin has been shown to possess multiple pharmacological activities such as anti-inflammatory and anti-cancer activities. In this study, we demonstrated that curcumin inhibits the proliferation of *Theileria*-transformed bovine leucocytes by promoting apoptosis and autophagy. The transcriptome analysis of curcumin treated cells showed that the genes involved in cell death and autophagy are also differentially regulated. We further elucidated the mechanism of action of curcumin on *Theileria* infected bovine cells. We found that curcumin induced the generation of reactive oxygen species (ROS) which activated caspase 8 and destabilized the mitochondrial membrane potential leading to the release of cytochrome c from mitochondria. This subsequently led to the activation of caspase 3 and PARP cleavage, finally leading to apoptosis in the infected cells. Furthermore, curcumin induced the process of autophagy which was characterized by the formation of acidic vesicular organelles, LC3B accumulation with lysosome inhibitor, E64d, and the presence of autophagosomes as visualized by transmission electron microscopy (TEM). Curcumin treatment suppressed the mTOR and increased the expression of autophagy-related proteins. We also found that N- acetylcysteine, an inhibitor of ROS, could rescue the infected cells from curcumin induced apoptosis and autophagy mediated cell death. Intriguingly, curcumin had no effect on uninfected bovine PBMCs. Altogether, these data suggest the therapeutic potential of curcumin against bovine tropical theileriosis.

## Introduction

Bovine tropical theileriosis is a tick-borne disease transmitted by *Hyalomma* spp., and is endemic to Southern Europe, North Africa and Asia^1^. It is caused by *Theileria annulata* which is an obligate intracellular protozoan parasite of order Piroplasmida. *T. annulata* has a complex life cycle comprising of two hosts^2^. After completion of sexual reproduction stages in the tick gut, *T. annulata* migrates to the acinar cells of tick salivary glands where it matures as sporozoites and is released in the saliva^2^. Upon entering the bovine bloodstream, sporozoites invade the monocytes, macrophages and/or B-cells. After parasite entry into the host leucocytes through a zippering mechanism, it clears the surrounding host membrane^3^ and alters several signaling pathways of the host, leading to the transformation of the host cells^4^.

*Theileria* infected bovine leucocytes have cancer hallmarks^5^. The homeostasis of various signaling pathways such as NF-κB, Ras-ERK, and PI3K-Akt get altered in the cancerous cells^6^. NF-κB is a transcription factor which plays a conserved role in apoptosis, proliferation, differentiation, and development^7^. The activation of NF-κB in cancer cells prevents apoptosis thus leading to cancer cell proliferation^8^. *Theileria* infected leucocytes have been shown to constitutively activate NF-κB^9^ leading to protection against apoptosis. Phosphoinositide 3-kinase (PI3-K)/Akt signaling plays a pivotal role in various signal transduction pathways. PI3-K/Akt signaling gets activated in response to growth factors and contributes to several cellular functions such as glucose metabolism, cell proliferation, apoptosis and transcription^10^. However, PI3-K/Akt pathway is aberrantly activated in human cancers leading to cellular transformation, cancer progression, and drug resistance^11^. *Theileria* transformed leucocytes activate Akt/PKB pathway in a parasite dependent manner but is shown not to be linked to NF-κB activation^12^. *Theileria* induces increased PI3-K activity in the infected B-lymphocytes which is required for continuous proliferation^13^. *Theileria* parasites activate the oncogene, c-Myc and promote the survival of infected B-lymphocytes^14^. The hypoxia inducible factor (HIF1) which is a master regulator of cellular and developmental O_2_ homeostasis has been shown to be activated in most of the cancers^15^. Further, anti-cancer effects of HIF1 inhibitors have been reported^16^. In theileriosis, the *T. annulata* has been shown to induce the HIF1a (a subunit of HIF1) activation^17^. Thus, there exist enough evidence that the *Theileria* parasite induces cancer-like phenotype in the host cells.

Curcumin (diferuloylmethane), a polyphenol extracted from the plant *Curcuma longa* (commonly known as turmeric), has been known to possess anti-inflammatory and anti-cancer properties^18,19^. Curcumin has been demonstrated to modulate the proliferation and cellular response of macrophages, natural killer cells and various other immune cell types^20,21^. Curcumin kills tumor cells by modulating several cell signaling pathways such as it inhibits activation of NF-κB leading to apoptosis in the tumor cells^22,23^. Further, curcumin induces apoptosis through the release of cytochrome c and inhibits Akt in renal cancer cells^24^. It is also considered that PKC, mTOR, and EGFR tyrosine kinase are the major upstream molecular targets for curcumin whereas c-jun, c-myc, cyclin dependent kinases, and Akt are the downstream targets^25^. Furthermore, clinical trials of curcumin on humans against various cancers have been encouraging^26–28^ (www.clinicaltrials.gov).

In the present study, we demonstrate for the first time that curcumin induces apoptosis in *Theileria* infected bovine leucocytes but not in uninfected bovine PBMCs. Further, we unveil the mechanism of action of curcumin in killing of the *Theileria* infected bovine leucocytes. We show that the curcumin treatment of *Theileria* infected cells leads to caspase 8 activation, the release of cytochrome c to the cytosol and finally the activation of caspase 3. We also demonstrated for the first time that curcumin treatment of *Theileria* infected cells induces autophagy pathway through mTOR inhibition. Finally, we showed that curcumin activates both the apoptosis pathway and autophagy by inducing oxidative stress which can be reverted back by using an anti-oxidant. This is first study showing therapeutic potential of curcumin against theileriosis.

## Materials and methods

### Chemicals and reagents

Ficoll paque plus, DMSO, curcumin, N- acetyl L- cysteine (NAC), and 2′,7′-Dichlorofluorescin diacetate (DCF-DA) were purchased from Sigma- Aldrich. Culture medium RPMI-1640, fetal bovine serum, and penicillin-streptomycin were obtained from Gibco (Life Technologies). Acridine orange purchased from Molecular Probes (Life Technologies). Caspase 3 fluorogenic substrate Ac (N-acetyl)-DEVD-AFC, caspase 3 inhibitor (Ac-DEVD-CHO), caspase 8 fluorogenic substrate Ac-IETD-AFC, caspase 8 inhibitor (Z-IETD-FMK) and antibodies against caspase 8 and cytochrome c were purchased from BD Pharmingen. Antibodies against β-tubulin, and Akt, were purchased from Santa Cruz Biotechnology. Antibodies against LC3A/B, Phospho-Akt, PARP, mTOR, Phospho-mTOR (Ser 2448), and IKKβ were purchased from Cell Signaling Technology. Antibodies against Bcl2 and pBad were obtained from Novus Biologicals. Secondary antibodies, anti-mouse IgG conjugated with HRP, and anti-rabbit IgG conjugated with HRP were purchased from Pierce. E64d was purchased from Cayman chemicals. mCherry-hLC3B-pcDNA3.1 was a gift from David Rubinsztein (Addgene plasmid # 40827).

### Ethical statement

Complete blood was collected from the external jugular vein of apparently healthy cattle by the trained veterinarian with the consent of the owner. All the experiments were performed in accordance with relevant guidelines and regulations.

### Establishment of parasite cell culture from a field isolate

Blood sample from *Theileria* infected cattle was collected in Vacutainer (BD, Pharmigen), from Anantapur district of Andhra Pradesh, India. The whole blood was processed to isolate the peripheral blood mononuclear cells (PBMCs) using standard density gradient method. Briefly, blood was diluted with PBS in 1:1(v/v) ratio and then layered onto Ficoll Paque Plus (GE healthcare) in 3:4 (v/v) ratio, centrifuged at 400 g for 40 min at 28 °C. After centrifugation, bovine PBMCs were collected and washed twice with PBS. The isolated PBMCs were then cultured in complete RPMI media-1640 (Gibco, Life technologies) (supplemented with 10% fetal bovine serum, 2 mM L-glutamine, 25 mM HEPES, 0.1% PenStrep) at 37 °C with 5% CO_2_^29^. The field isolate thus obtained was named as Ana2014. Uninfected PBMCs from apparently healthy cattle (negative for *Theileria* infection) were also isolated using the standard protocol as mentioned above.

### Curcumin treatment to Ana2014 cells and cell viability assay

A stock solution of curcumin (100 mM) was prepared in DMSO and stored at 4 °C. For each experiment, curcumin was diluted with complete RPMI-1640 medium to the concentrations indicated so that a final DMSO concentration was less than 0.1% (v/v). Cells were seeded at 3 × 10^5^ cells/ml and treated with an appropriate concentration of curcumin after six hours of incubation. Trypan blue dye exclusion assay was performed after 12, 24, and 36 h of curcumin treatment and inhibition percentage was calculated using the formula,

Inhibition percentage = [(number of live cells in control−number of live cells in treated)/number of live cells in control] × 100.

### Curcumin uptake

After curcumin treatment (20 µM), 3 × 10^6^ cells were taken and washed twice with PBS. The cells were centrifuged at 1000 g for 5 min at room temperature and the cell pellet was resuspended in 300 μl of methanol. The cells were sonicated and centrifuged at 13000 × *g* for 15 mins at room temperature. The supernatant was collected and absorbance in the range of 300–700 nm using UV/visible spectrophotometer was analyzed. Curcumin uptake by the cells was estimated by plotting standard curve.

### Annexin V-FITC/ propidium iodide assay and Terminal deoxynucleotidyl transferase (TdT) mediated dUTP nick-end labeling (TUNEL) assay

Annexin V-FITC/propidium iodide (PI) assay was carried out according to the manufacturer (BD Pharmingen) recommended procedure. Briefly, 1 × 10^6^ cells were collected after curcumin treatment and washed with PBS. The cells were centrifuged at 400 g for 5 min at room temperature and were re-suspended in 100 µl of binding buffer containing 5 µl of annexin V-FITC and 5 µl of propidium iodide. The cells were analyzed for annexin V-FITC/PI staining after incubation for 15 min at room temperature in the dark.

For Terminal deoxynucleotidyl transferase (TdT) mediated dUTP nick-end labeling (TUNEL) assay, cells were labeled by catalytically incorporating FITC-dUTP at the 3′- hydroxyl ends of the fragmented DNA by enzyme terminal deoxynucleotidyl transferase (TdT) using APO-DIRECT kit (BD Pharmingen). Staining procedures were performed according to the instructions provided in the kit.

For both the Annexin V-FITC/propidium iodide and TUNEL assay, a total of 10,000 events were acquired on a flow cytometer (BD LSRFortessa, BD Biosciences) and analyzed using FlowJo software.

### Library Preparation, Illumnia sequencing, and gene ontology studies

The Ana2014 cells were treated with 20 μM curcumin for 24 h. Total RNA was extracted from untreated and 24 h curcumin treated Ana2014 cells using RNAiso Plus (TaKaRa, Japan) followed by treatment with DNase I. Whole transcriptome sequencing was carried out at Genotypic Technology Pvt. Ltd., Bangalore, India. RNA purity, concentration and integrity values were assessed using Bioanalyzer 2100 system (Agilent Technologies, CA, USA). Sequencing libraries were constructed following NEBNext® Ultra™ Directional RNA Library Prep Kit protocol and then processed by Illumina HiSeq Paired-end sequencing. Raw read data was run through quality control metrics using FastQC (http://www.bioinformatics.babraham.ac.uk/projects/fastqc/). Sequence reads were aligned with the *Bos taurus* genome (UMD3.1 build, Ensembl.org) and *Theileria annulata* reference genome (ftp://ftp.sanger.ac.uk/pub/project/pathogens/T_annulata) using HISAT2. The quantification of the transcripts was performed using Cufflinks^30^. Cuffdiff was used to calculate the differentially expressed transcripts and categorized them into upregulated or down-regulated based on log_2_fold change values^31^. Genes with log2fold change values below −1.5 and above 1.5 were considered as downregulated and upregulated respectively, compared to the control. These genes were further analyzed for gene ontology based on biological process (GO-BP) using the Generic Gene Ontology Term Finder^32^.

### Validation of differentially regulated genes by quantitative RT-PCR

The Ana2014 cells were cultured with and without 20 µM curcumin for 24 h. Total RNA was isolated from both curcumin untreated and treated cells using Nucleospin RNA plus (Macherey-Nagel, Germany) according to manufacturer instructions. RNA concentration was quantified using NanoDrop 1000 Spectrophotometer (Thermo Fisher Scientific Inc., USA). Samples with A260/280 ratio between 1.9 and 2.1 and A260/230 ratio greater than 2.0 were used for cDNA synthesis. One microgram of RNA was taken for cDNA preparation using PrimeScript 1st strand cDNA Synthesis Kit (TaKaRa, Japan) according to the manufacturer’s instructions. The cDNA was diluted 10-fold with deionised water and used as template for quantitative real time PCR (qRT-PCR). qRT- PCR was performed using SYBR Premix Ex Taq™ (Tli RNaseH Plus) (TaKaRa, Japan) and 7500 Real Time PCR detection system (Applied Biosystem, USA). The reaction mixture (10 µl) contained 5 µl 2× SYBR Premix Ex Taq™, 0.2 µM each of forward and reverse primers, 2 µl cDNA and 0.2 µl ROX reference dye II. The thermal cycle conditions used were as follows: 95 °C for 10 min followed by 40 cycles of 95 °C for 15 s, annealing at variable temperature (primer pair dependent) for 30 s, 72 °C for 10 s and finally melt curve conditions pre-set in 7500 Real Time PCR detection system. TBP (TATA box binding protein) and PPIA (Peptidylprolyl isomerase A) were used as endogenous controls. The BoTBP and BoPPIA are found to be the stable reference genes for bovine host (unpublished data). The expression level of upregulated and downregulated genes was calculated using the 2^−ΔΔCt^ method by normalizing with the geometrical mean of the Ct values of BoTBP and BoPPIA. The primer sequences of the genes are listed (Table S1).

### Determination of caspase 3 and caspase 8 activity

Total protein from curcumin treated and untreated cells were extracted in ice-cold cell lysis buffer (10 mM Tris HCl, 10 mM NaH_2_PO_4_/NaHPO_4_ pH 7.5, 130 mM NaCl, 1% Triton X-100, 10 mM sodiumpyrophosphate) by incubating on ice for 15 mins. After incubation, cells were briefly sonicated and centrifuged at 13,000 × *g* for 10 min at 4 °C. The supernatant was used for assay after quantifying protein concentration through BCA assay kit (Pierce, Thermo Scientific). A 50 µg of total protein of curcumin treated and control samples each were added to the reaction mixture containing either caspase 3 substrate (Ac (N-acetyle)-DEVD-AFC) with and without caspase 3 inhibitor (Ac-DEVD-CHO) or caspase 8 substrate (Ac-IETD-AFC) with and without caspase 8 inhibitor (Z-IETD-FMK) in caspase reaction buffer (40 mM HEPES pH-7.5, 20% glycerol, 4 mM dithiothitol). The release of AFC was detected using multimode plate reader (PerkinElmer EnSpire) with an excitation wavelength of 400 nm and an emission wavelength of 505 nm. The relative fluorescence units of curcumin treated samples with respect to untreated samples were plotted. Further, the inhibition of specific activity of caspase 3 and caspase 8 were determined by their respective inhibitors.

### Western blot

Cell pellets of different experimental conditions were lysed in RIPA buffer (50 mM Tris–HCl pH-7.4, 1% NP-40, 0.5% sodium deoxycholate, 0.1% SDS, 150 mM NaCl, 2 mM EDTA, 50 mM sodium fluoride, 0.2 mM sodium orthovanadate, 1 mM PMSF, 1 mM leupeptin) by incubating on ice for 30 min and then by brief sonication. The cell debris was removed by centrifugation at 13,000 × *g* for 10 min at 4 °C and supernatant was collected. Protein concentration was determined using BCA assay kit (Pierce, Thermo Scientific). Laemmli buffer (5 × ) was added to each cell lysate. The lysate was denatured by incubating at 95 °C for 5 min. Cell lysates (50 µg per lane) were resolved by SDS-PAGE (12% or 10% (w/v) polyacrylamide). Proteins were then electro-transferred onto PVDF membranes. The membranes were blocked with 5% non-fat milk or 5% BSA in TBST (TBS and 0.1% Tween) for 1 h at room temperature and incubated with primary antibodies for overnight at 4 °C. After washing thrice with TBST the membrane was incubated with the relevant secondary antibodies for 1 h at room temperature. After incubation, the membrane was washed thrice with TBST and chemiluminescent signals were captured using SuperSignal West Pico Chemiluminescent Substrate (Thermo Scientific) in G: BOX Chemi imaging system (Syngene). The protein band density was quantified using the ImageJ software (Version 1.51k; NIH, Bethesda, MD, USA), with β-tubulin as a loading control. The relative intensity of each band was normalized to the band of β-tubulin respectively. All experiments were conducted in triplicate.

### Preparation of cytosolic and mitochondrial fraction

In total 1 × 10^7^ cells (curcumin treated and untreated) were washed once with PBS and were re-suspended in 500 µl of mitochondrial isolation buffer (10 mM HEPES- KOH, pH 7.2, containing 1.5 mM MgCl_2_, 1 mM EDTA, 1 mM EGTA, 0.21 M sucrose, 70 mM mannitol, 1 mM PMSF, 1 mM leupeptin)^33^. Cells were incubated on ice for 60 min with frequent taping. The cellular suspension was homogenized with a glass dounce homogenizer with 40 times up and down passes of the pestle. The homogenate was centrifuged at 1000 × *g* for 10 min at 4 °C to remove nuclei and intact cells. The supernatant was centrifuged at 13,000 × *g* for 15 min at 4 °C to remove mitochondria and other cell organelles. The resulting supernatant (cytosolic fraction) was taken for western blotting.

### Acridine orange staining

Cells (curcumin treated and untreated) were pelleted down and washed once with PBS. Cells were then stained with 5 µg/ml acridine orange at 37 °C for 15 min in dark, mounted on poly-lysine coated slides. The images were collected immediately using confocal microscope (Leica SP8, Leica Microsystems) and processed using LAS X software.

### Fluorescent measurement of ROS

DCF-DA was used to detect ROS production in curcumin treated cells. Briefly, 3 × 10^5^ cells treated with or without 10 mM NAC for two hours before curcumin treatment. After washing twice with PBS, the cells were incubated with 20 µM DCF-DA in PBS at 37 °C and 5% CO_2_ in dark for 20 min. Fluorescence intensities were recorded using a flow cytometer (BD LSRFortessa, BD Biosciences).

### Transfection

Plasmid (mCherry-hLC3B-pcDNA3.1) was transfected into the Ana2014 cells using Lonza nucleofection protocol following DS103 program and with SF solution. 1 × 10^6^ cells were used per transfection with 2 μg plasmid DNA in 82 µl of SF solution and 18 µl of supplement. The cells were treated with curcumin 24 h post-transfection. The cells were visualized under confocal microscope (Leica SP8, Leica Microsystems) and the images were processed using LAS X software.

### Transmission electron microscopy (TEM)

Curcumin untreated and treated cells were fixed in 2.5% glutaraldehyde in 0.1 M phosphate buffer (pH 7.2) for 24 h at 4 °C, and washed 4 times each for 45 min with PBS. The cells were then post fixed in 1% aqueous osmium tetroxide for 2 h and washed six times with deionized distilled water for 45 min in each wash. The samples were dehydrated with series of graded alcohols, infiltrated and embedded in araldite 6005 resin or spurr resin^34^. The cells were then incubated at 80 °C for 72 h for complete polymerization. Ultra-thin (60 nm) sections were made with a glass knife on ultra-microtome (Leica Ultra UCT-GA-D/E-1/00), mounted on copper grids and stained with saturated aqueous urenyl acetate (UA), and counterstained with Reynolds lead citrate (LC). Transmission electron microscopy was carried out with JEM2100 (Jeol, Japan) transmission electron microscope facility at the CSIR-Centre for Cellular and Molecular Biology (CSIR-CCMB), Hyderabad, India.

### Statistical analysis

All experiments (except RNAseq experiment) were performed at least thrice. The results are shown as the mean values ± standard deviation (S.D.). The statistical tests were performed with the software Graphpad Prism (Version 7.04). The data were statistically analyzed by *t*-test. For all the tests, *p* < 0.05 was considered significant. In case of RNAseq experiment only one sample each of control (untreated) and curcumin treated were used.

## Results

### Isolation and characterization of Ana2014 cells: a field isolate

The isolated PBMCs collected from *Theileria* infected bovine which survived after 4–5 passages in complete RPMI media were named as Ana2014 (unpublished work). Only *Theileria* infected cells have the potential to proliferate indefinitely in culture conditions. The presence of *Theileria annulata* in Ana2014 was confirmed using AccuPower *Theileria* PCR kit (Bioneer, Korea) (Figure S2a). Also, PCR with *Theileria* specific genes TA18945 (TaPIN), TA19600, and TA13185 confirmed the presence of *Theileria* parasites in Ana2014 cells (Figure S2b).

### Curcumin inhibits the proliferation of Ana2014 cells

**Fig. 1:**
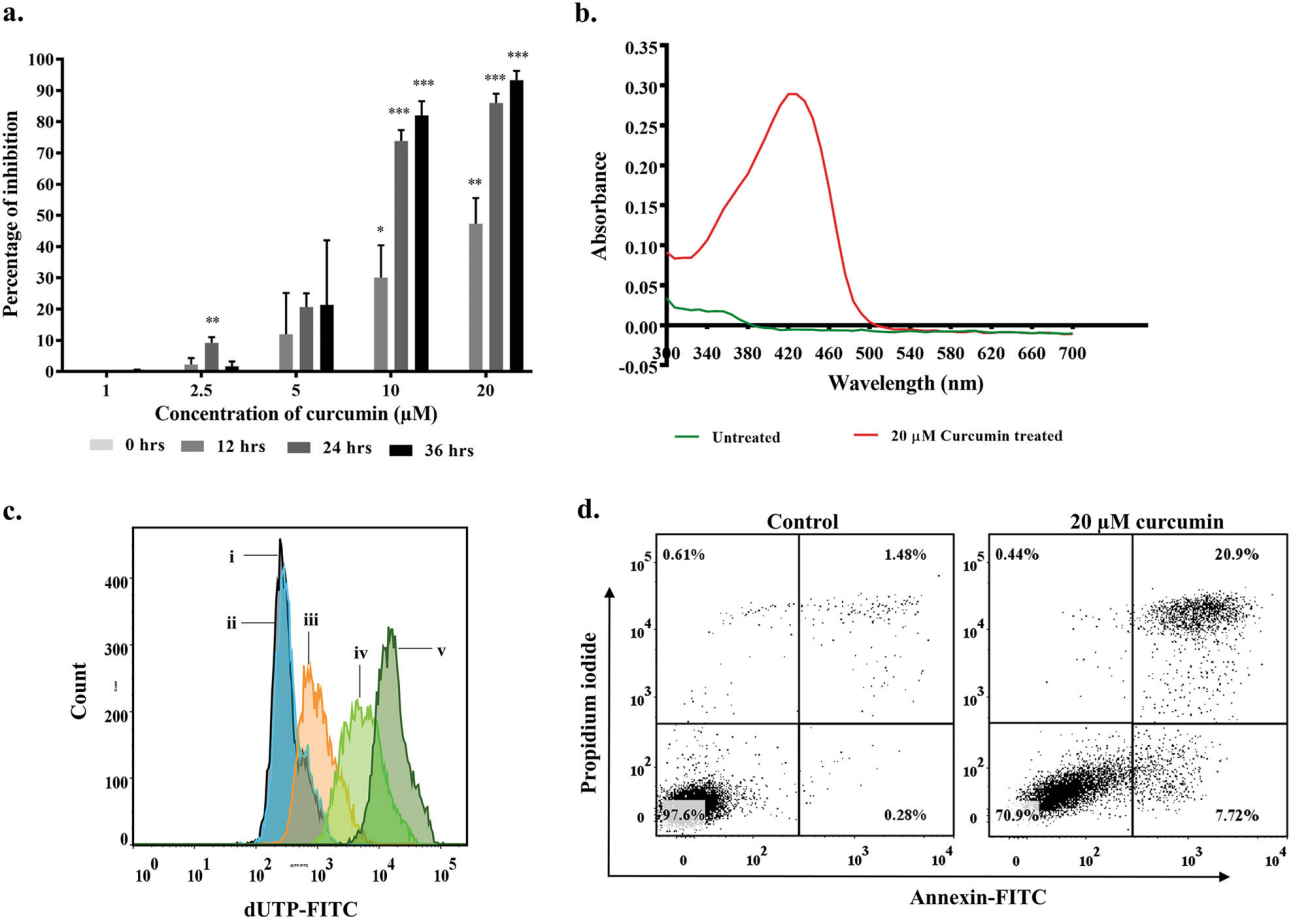
Cellular uptake of curcumin and induction of cell death in Ana2014 cells. **a** percentage of growth inhibition upon treating infected cells with various concentrations (1, 2.5, 5, 10 and 20 µM) of curcumin at various time points (12, 24 and 36 h), **b** Absorbance of curcumin in methanolic extract of Ana2014 cells. Red line indicates the absorbance in the curcumin treated cells and green line indicates the absorbance in untreated cells, **c** TUNEL assay representing DNA fragmentation induced by curcumin. (i) unstained, (ii) untreated, (iii) 20 µM, (iv) 40 µM and (v) 80 µM, **d** Annexin V FITC- PI staining of untreated and 20 µM curcumin treated Ana2014 cells for 24 h, showing the percentage of live, early apoptotic, apoptotic and necrotic cell population. *N* = 3. Data are presented as mean ± SD. * represents *p* < 0.05, ** represents *p* < 0.01, and *** represents *p* < 0.001, compared with the untreated group

To examine the effect of curcumin on proliferation of Ana2014 cells, the cells were treated with different concentrations (1 µM, 2.5 µM, 5 µM, 10 µM, and 20 µM) of curcumin for 12 h, 24 h, and 36 h. The viability of the cells was assessed through trypan blue dye exclusion assay after every 12 h which showed that curcumin inhibited the growth of Ana2014 cells in a dose-dependent and time-dependent manner (Fig. 1a). However, no cell death was observed when uninfected PBMCs isolated from healthy cattle were treated with curcumin (20 µM) for 24 h (Figure S1a). This suggests that curcumin specifically kills *Theileria* infected cells.

Since there is a linear relationship between the curcumin concentration and absorbance at 428 nm (Figure S1b), we estimated actual curcumin uptake by Ana2014 cells by the absorbance measurement at 428 nm of methanolic extract of cell lysates. After incubating Ana2014 cells with 20 µM curcumin for 24 h, 1.16 µg/million/ml of curcumin was found within the cells (Fig. 1b).

### Curcumin treatment of Ana2014 cells leads to differential expression of genes involved in autophagy and cell death

Whole transcriptome sequencing carried out to evaluate the influence of curcumin treatment on Ana2014 cells was submitted to the SRA database with accession number-GSE119138. The transcriptome analysis showed total 12487 transcripts of *Bos taurus*, on an average, in both untreated and curcumin treated cells. Cuffdiff analysis showed that 795 transcripts were upregulated and 2131 transcripts were downregulated after curcumin treatment. The differentially regulated genes data was validated by qRT-PCR. Eight transcripts were randomly chosen from each upregulated and downregulated dataset. The qRT-PCR analysis showed similar regulation as obtained from the RNA-seq experiment data (Figure S4).

**Fig. 2:**
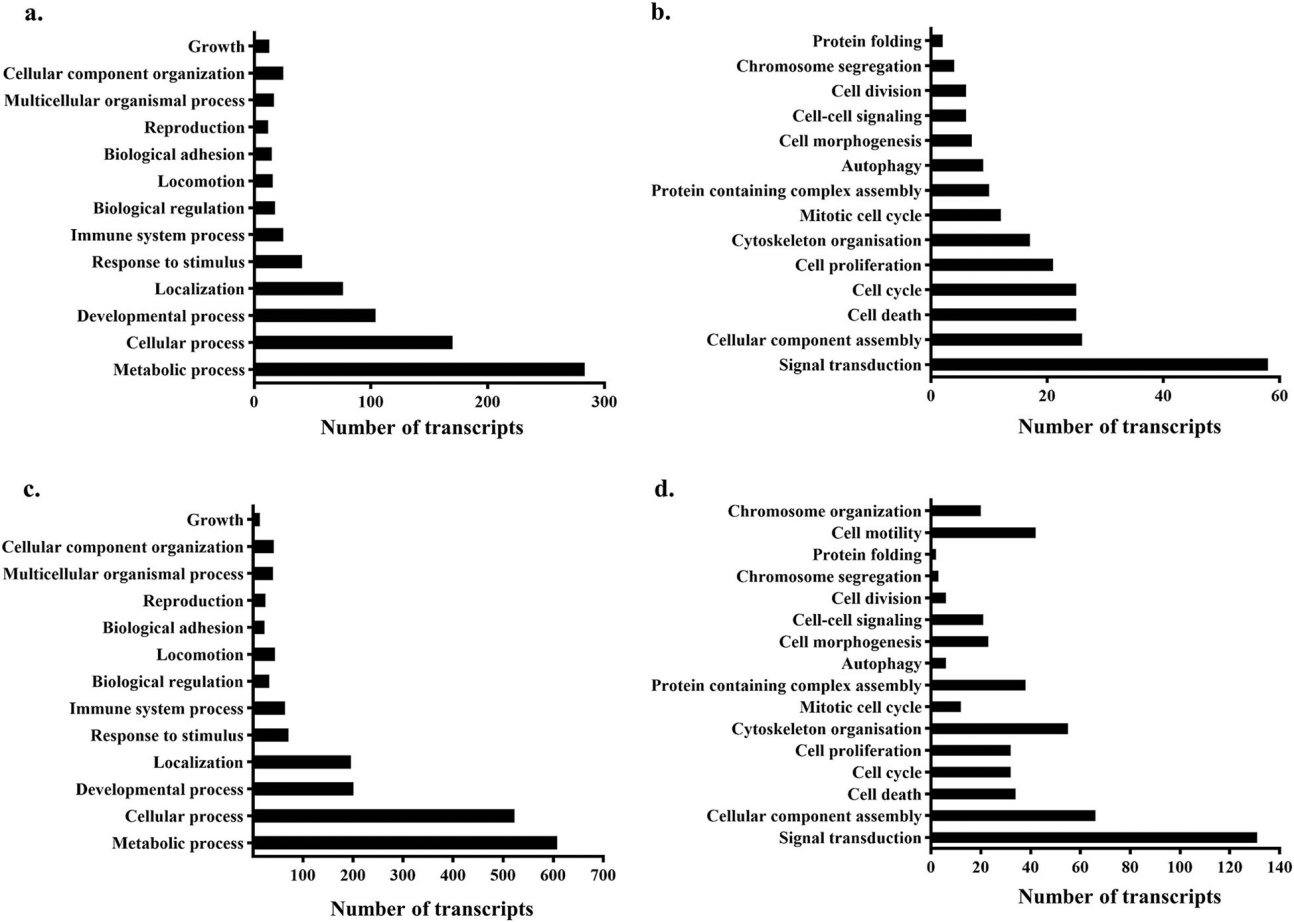
Transcriptomic changes induced by curcumin in Ana2014 cells. **a** Gene Ontology-Based on Biological Process (GO-BP) analysis of upregulated genes (log2fold change > 1.5), **b** Analysis of upregulated transcripts involved in cellular process, **c** GO-BP analysis of downregulated genes (log2fold change < −1.5), **d** Analysis of downregulated transcripts involved in cellular process

The log2fold change values with the cutoff of above 1.5 and below −1.5 were considered as upregulated and downregulated respectively. In total 443 transcripts were found to have >1.5 log2fold change value and 933 transcripts were found to have less than −1.5 log2fold change value. The heap map of these differentially expressed genes is shown in Figure S5. The GO-BP analysis showed that out of the various biological processes such as metabolic processes, cellular processes, developmental processes, immune system processes, biological regulation etc., majority of these differentially expressed genes were involved in the metabolic processes and cellular processes, both in the upregulated and downregulated transcripts (Fig. 2a and 2c). Further, under the category of cellular processes, genes involved in cell death and autophagy were observed for both upregulated and downregulated transcripts (Fig. 2b and 2d) which suggests that cells might activate autophagy and apoptotic pathways upon curcumin treatment.

### Curcumin induces apoptosis in Ana2014 cells

To explore the status of DNA fragmentation upon curcumin treatment, the curcumin treated (20, 40 and 80 µM) and untreated cells were labeled by catalytically incorporating FITC-dUTP at the 3′- hydroxyl ends of the fragmented DNA by enzyme terminal deoxynucleotidyl transferase. The results of the flow cytometric analysis showed increase in the DNA fragmentation with increasing concentration of curcumin (Fig. 1c). Furthermore, the apoptotic cell population was determined quantitatively by annexin V-FITC/PI apoptosis detection kit after incubating cells with 20 µM curcumin for 24 h. FACS analysis showed an increase in the apoptotic cell population with curcumin treatment (Fig. 1d, and Figure S3).

### Curcumin induces caspase-dependent apoptosis in Ana2014 cells

**Fig. 3:**
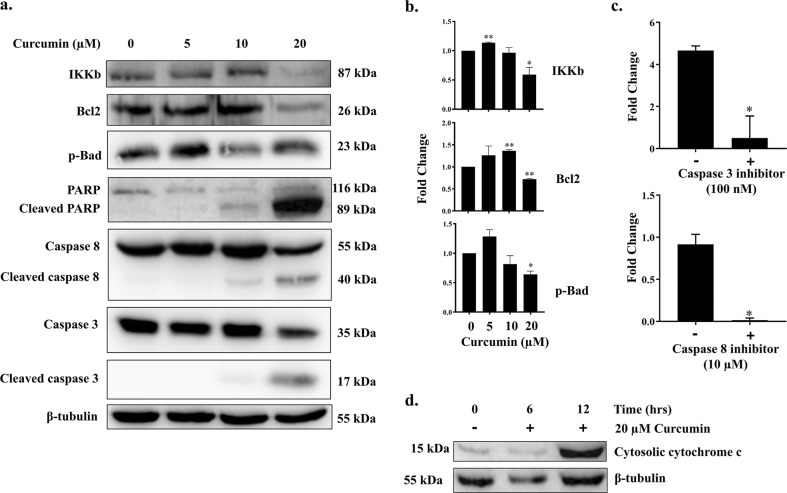
Curcumin induces caspase activation in Ana2014 cells. **a** Western blot presenting the cleaved forms of caspase 3, caspase 8 and PARP upon curcumin treatment at various concentrations (5, 10, and 20 µM) for 24 h. Change in the expression level of the IKKb, Bcl2, and p-Bad upon curcumin treatment at various concentrations (5, 10, and 20 µM) for 24 h, **b** fold change in the expression of IKKb, Bcl2, and p-Bad upon curcumin treatment after normalization of western blot data, **c** After curcumin treatment, the activities of caspase 3 and caspase 8 were recorded. The activity of caspase 3 and caspase 8 were shown in the presence and absence of their inhibitors after 20 µM curcumin treatment. **d** Western blot showing the amount of cytochrome c present in cytoplasm after 20 µM curcumin treatment at different time points (6 h and 12 h). *N* = 3. Data are presented as mean ± SD. * represents *p* < 0.05, ** represents *p* < 0.01, and *** represents *p* < 0.001, compared with the untreated group

Caspases have been found to be importantly involved in apoptosis. We thus examined the cleaved forms of caspase 8 and caspase 3 by western blot after treating cells with 5, 10, and 20 µM curcumin for 24 h. Cleaved caspase 3 was detected only with 20 µM of curcumin treatment whereas cleaved caspase 8 was observed with both 10 µM and 20 µM curcumin treatment (Fig. 3a). The activities of caspase 3 and caspase 8 were also measured. The increase in the activity of both caspase 3 and caspase 8 was observed after 20 µM curcumin treatment to Ana2014 cells which was abolished in the presence of their inhibitors (Fig. 3c). Further, we examined the fate of PARP which is present at the downstream of caspase pathway. The cleaved form of PARP was observed upon the treatment of Ana2014 cells with 20 µM curcumin, suggesting the induction of caspase-dependent apoptosis in treated cells (Fig. 3a).

### Curcumin induces cytochrome c release from mitochondria in Ana2014 cells

The decrease in the level of Bcl2 is required for the activation of apoptosis. We also observed the decrease in the Bcl2 level upon curcumin treatment of the Ana2014 cells (Fig. 3a, b). The decrease in Bcl2 levels in the cells promote the release of cytochrome c from mitochondria, which subsequently leads to the activation of caspases. The Ana2014 cells treated with 20 µM curcumin for 6 h and 12 h revealed that there was the release of cytochrome c in the cytoplasm of Ana2014 cells in time-dependent manner (Fig. 3d). This suggests that curcumin also activates the intrinsic apoptotic pathway in Ana2014 cells.

### Curcumin induces autophagy in Ana2014 cells

**Fig. 4:**
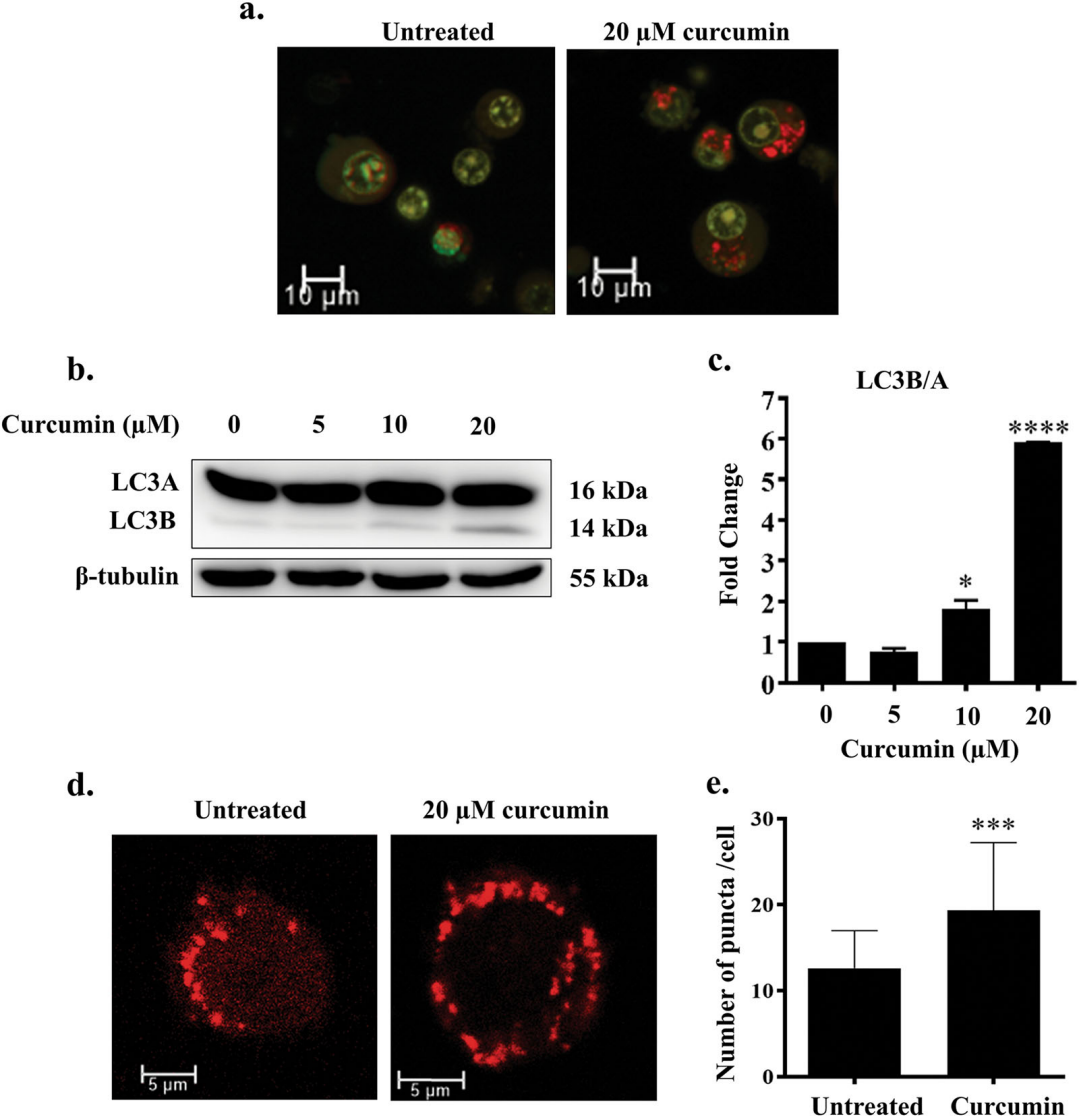
Curcumin induces autophagy pathway in Ana2014 cells. **a** Confocal microscopy of Ana2014 cells treated with curcumin (20 µM) showing the accumulation of many acridine orange (AO) positive acidic vesicles, **b** Western blot analysis of Ana2014 cells treated with various concentrations (5, 10, and 20 µM) of curcumin showing the conversion of LC3A to LC3B, **c** Fold change in expression of LC3B/A after normalization of western blot data. *N* = 3. Data are presented as mean ± SD. * represents *p* < 0.05, ** represents *p* < 0.01, and *** represents *p* < 0.001, compared with the untreated group, **d** Ana2014 cells transiently transfected with mCherry-hLC3B-pcDNA3.1 were treated with 20 µM curcumin. mCherry-hLC3B-pcDNA3.1 puncta were observed by confocal microscope, **e** significant increase in the number of puncta per cell upon curcumin treatment

**Fig. 5:**
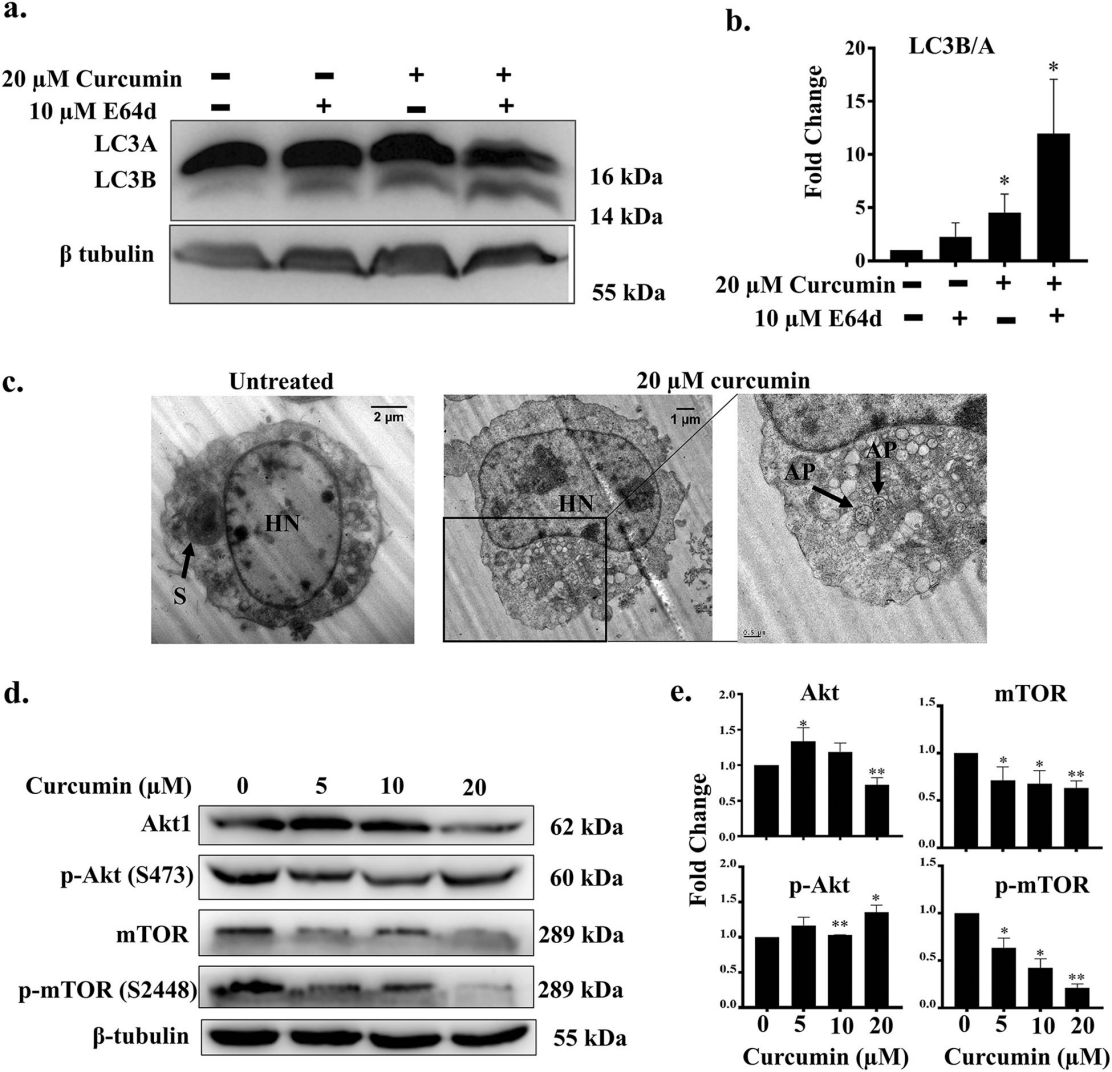
Effect of E64d on LC3B/A levels in curcumin treated Ana2014 cells. **a** Western blot showing stabilization of LC3B in Ana2014 cells by E64d treatment prior to curcumin treatment, **b** Fold change in the expression of LC3B/A after E64d and curcumin treatment. *N* = 3. Data are presented as mean ± SD. * represents *p* < 0.05, ** represents *p* < 0.01, and *** represents *p* < 0.001, compared with the untreated group, **c** TEM images of control and curcumin treated cells. Enlarged section in the image locating the autophagosomes within the curcumin treated cell, S=Schizont, HN=Host nucleus, AP=Autophagosome **d** Western blot analysis of Ana2014 cells treated with various concentrations (5, 10, and 20 µM) of curcumin showing the changes in the expression levels of Akt, p-Akt, mTOR and p-mTOR, **e** Fold change in expression of Akt, p-Akt, mTOR, and p-mTOR proteins after normalization of western blot data. *N* = 3. Data are presented as mean ± SD. * represents *p* < 0.05, ** represents *p* < 0.01, and *** represents *p* < 0.001, compared with the untreated group

The cells were stained with a lysosomal dye acridine orange (AO) to visualize the autolysosomes in the Ana2014 cells. The AO binds to dsDNA and emits green fluorescence while it emits red fluorescence when it is bound to ssDNA or RNA. It emits orange fluorescence inside acidic vacuoles. We observed the increase in orange fluorescence after curcumin treatment (Fig. 4a) which indicates the increase in autolysosomes upon curcumin treatment. Next, we examined the levels of LC3B after curcumin treatment. The western blotting showed that curcumin stimulates the dose dependent conversion of LC3B (Fig. 4b, c). To further confirm the increase in the formation of autophagosomes, we transfected Ana2014 cells with mCherry-hLC3B-pcDNA3.1 plasmid. We observed a significant increase in the LC3B puncta formation (Fig. 4d, e) suggesting that there was an increase in the number of autophagy vacuoles. Since the lysosomal turnover, but not a cellular level of endogenous LC3, is a marker for autophagy^35^, we further assessed the levels of LC3B with respect to LC3A in presence of E64d, an inhibitor of lysosomal proteases, with curcumin treatment. We found that there is accumulation of LC3B upon treatment with E64d prior to curcumin treatment which confirm that there is increase in lysosomal turnover of LC3B in Ana2014 cells (Fig. 5a, b). Finally, we examined the formation of autophagosomes through TEM where autophagosomes were clearly seen in Ana2014 cells after curcumin treatment (Fig. 5c). These results confirm that curcumin induces autophagy in Ana2014 cells.

To further evaluate the signaling pathway underlying curcumin induced autophagy, the activation status of mTOR and its upstream regulator Akt were determined by western blotting. We found that the expression levels of phosphorylated mTOR was significantly decreased in the curcumin treated Ana2014 cells suggesting the activation of the process of autophagy (Fig. 5d, e). However, we were surprised to observe slight increase in the phosphorylated Akt (Fig. 5d, e).

### Curcumin induces reactive oxygen species mediated apoptosis and autophagy in Ana2014 cells

**Fig. 6:**
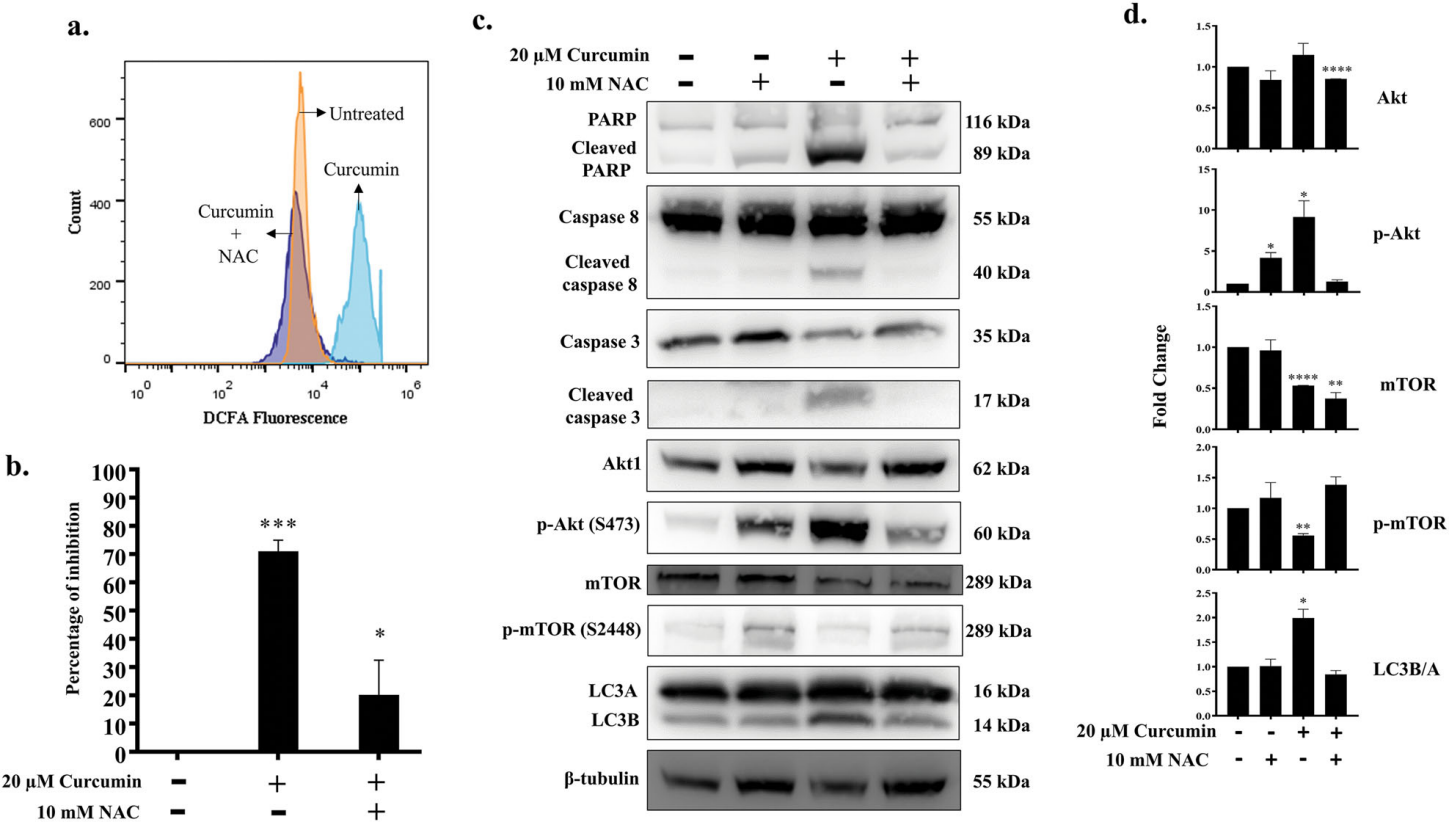
Curcumin generates ROS, associated with cell death. **a** detection of the levels of ROS by DCFH-DA fluorescence upon curcumin treatment and both curcumin and NAC treatment, **b** percentage of growth inhibition upon treating Ana2014 cells with curcumin (20 µM for 24 h) alone and in the presence of NAC, **c** Western blot showing the expression levels of apoptosis and autophagy-related proteins after curcumin treatment with and without inhibition of ROS using NAC, **d** Fold change in expression of apoptosis and autophagy-related proteins after curcumin treatment with and without inhibition of ROS using NAC. *N* = 3. Data are presented as mean ± SD. * represents *p* < 0.05, ** represents *p* < 0.01, and *** represents *p* < 0.001, compared with the untreated group

Reactive Oxygen Species (ROS) activate several signaling pathways that leads to apoptosis^36^. To investigate the ability of curcumin to generate ROS in Ana2014 cells we used the specific oxidation sensitive fluorescent dye DCFH-DA. DCFH-DA is a cell permeable dye which gets oxidized intracellularly in presence of the ROS and forms a fluorescent product dichlorofluorescein (DCF). DCF can be monitored by fluorescence based techniques. Increased fluorescence of DCF was observed after curcumin treatment to Ana2014 cells (Fig. 6a). However, addition of anti-oxidant, NAC, prior to curcumin treatment was found to decrease the DCF fluorescence in Ana2014 cells, suggesting there was indeed an increase in the ROS production after curcumin treatment. Further, we found that the addition of NAC prior to curcumin treatment leads to the decrease in the curcumin induced death in Ana2014 cells (Fig. 6b). So, next we studied the effect of ROS in the regulation of curcumin induced apoptosis and autophagy in Ana2014 cells. We observed that decrease in ROS could revert back the activation of caspase 3, caspase 8 and LC3B/A levels (Fig. 6c, d). The upstream regulator in autophagy such as phosphorylated Akt was found to be decreased while phosphorylated mTOR was found to be increased suggesting that oxidative stress plays an important role in curcumin mediated cell death in the Ana2014 cells (Fig. 6c, d).

## Discussion

*Theileria annulata* and *Theileria parva* are the two parasites which have the potential to transform the host cells. *Theileria* infected bovine leucocytes have cancer hallmarks such as hyper-proliferation, immortality, deregulation of cellular energetics, activation of invasion etc^5^. Curcumin is known to have anti-cancer activity with pharmacological safety^27,37^. In order to evaluate the potential of curcumin to control the proliferation of *T. annulata* infected cells, we treated Ana2014 cells with curcumin and observed that the proliferation was inhibited both by time and dose-dependent manner. Curcumin interacts with the cellular membrane and is shown to accumulate in the membranous structures such as plasma membrane, endoplasmic reticulum and nuclear envelope due to its lipophilic nature^38^. Phase I clinical trials, with curcumin as an anti-cancer agent, in humans, showed that curcumin exhibits limited bioavailability due to poor absorption, rapid metabolism, and rapid systemic elimination^39^. For this reason, we estimated the quantitative uptake of curcumin at 20 µM. We found that Ana2014 cells could take up almost 1/7th of the externally provided curcumin (20 µM). Further, curcumin has been shown to kill tumor cells by modulating several cell signaling pathways^22^. Thus, we performed the whole transcriptome analysis of Ana2014 cells under curcumin treated and untreated conditions to observe the global change in the transcriptome profile and their effect on various signaling pathways. The gene ontology analysis of differentially expressed genes based on biological processes showed that the genes involved in cell death and autophagy were differentially regulated upon curcumin treatment along with other biological processes.

**Fig. 7:**
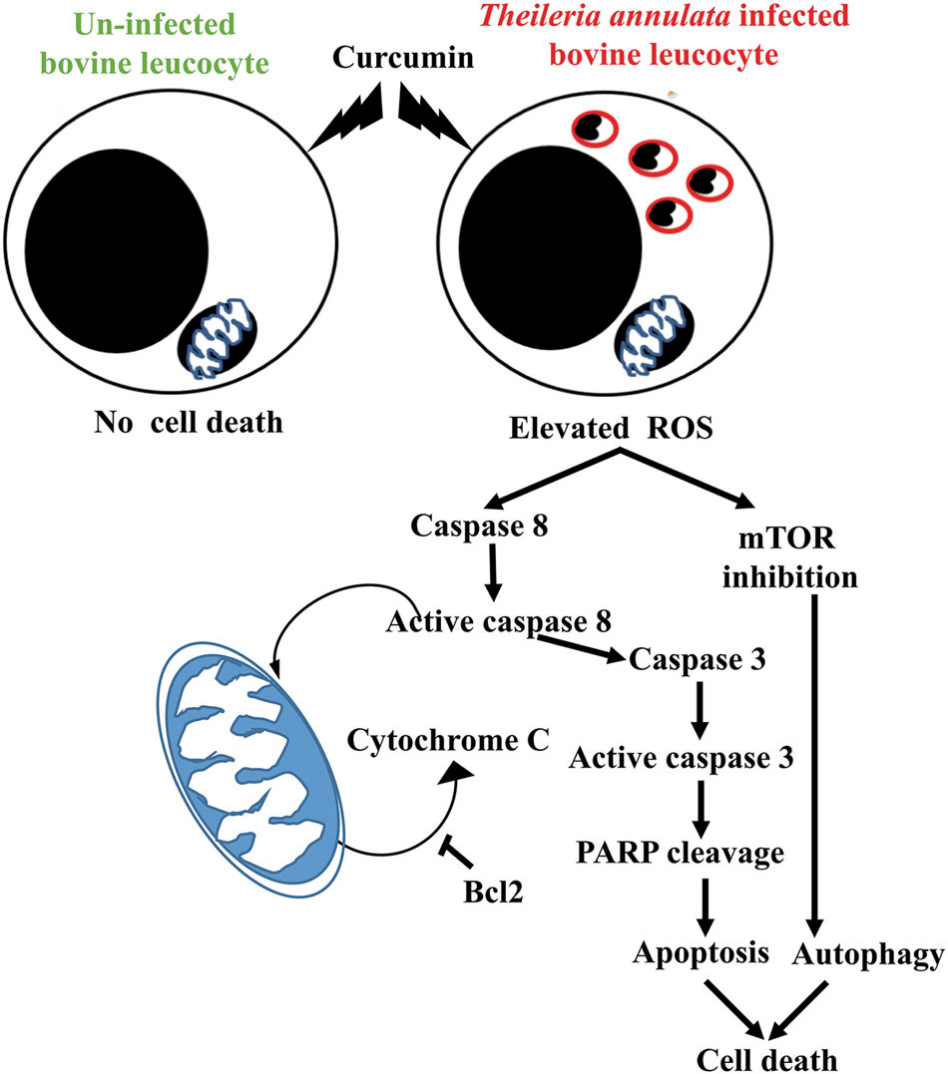
Schematic representation of the mechanism of action of curcumin on the Ana2014 cells and uninfected bovine PBMC. Curcumin treatment increases ROS levels in the Ana2014 cells which lead to cytochrome c and caspase mediated apoptosis. Increased ROS levels induce autophagy through inhibition of mTOR in Ana2014 cells, whereas curcumin has no effect on uninfected bovine PBMC

Curcumin has been shown to induce apoptosis in various cancer cells^40–42^. DNA fragmentation is a biochemical hallmark of apoptosis^43^. Hence, we carried out TUNEL assay and found that curcumin treatment leads to DNA fragmentation in Ana2014 cells in dose-dependent manner. Further, there was a significant increase in apoptotic cell number assessed through annexin V/propidium iodide staining.

Bcl-2 protein has the ability to inhibit apoptosis by preventing the efflux of cytochrome c from the mitochondria^44^. The release of cytochrome c from mitochondria is essential for the activation of caspases which are required for the commencement of apoptosis^45,46^. The overexpression of Bcl-2 also blocks caspase 8 and caspase 3 activation thus prevents apoptosis^47^. Our data is consistent with the previous studies as curcumin treatment of Ana2014 cells resulted in the decreased expression of Bcl-2 which resulted in time-dependent release of cytochrome c to the cytoplasm and activation of caspase 8, caspase 3 and PARP cleavage in Ana2014 cells. These observations suggest that curcumin induces caspase dependent apoptosis in Ana2014 cells. Also, IkB kinase β (IKKβ), which a catalytic subunit of IkB kinase complex (IKK), is required for the prevention of apoptosis^48^. So, next we analyzed the changes in the levels of IKKβ after curcumin treatment in Ana2014 cells and we found that curcumin treatment caused a decreased level of IKKβ.

Oxidative stress plays a vital role in mediating apoptosis^49^. Previous studies have shown that curcumin generates ROS in several cancer cells and triggers apoptosis^50,51^. In the present study, we found that curcumin induces oxidative stress by generating reactive oxygen species within the cell. And when we pre-treated the cells with N-acetyl-L-cysteine (NAC), a ROS inhibitor, cells were rescued from curcumin induced death and there was neither activation of caspases nor PARP cleavage. These findings confirm that curcumin induces oxidative stress-mediated apoptosis in *Theileria* infected bovine leucocytes.

Autophagy is an evolutionarily conserved, lysosomal degradation process. It is distinct from apoptosis where ubiquitinated proteins and damaged organelles are collected in the autophagosome, which finally fuses with a lysosome. In our study, curcumin treatment was found to promote increase the in acidic vacuoles (lysosome) visualized by AO staining. Autophagy has been shown to have roles both in cell survival and in cell death^52^. Microtubule-associated protein 1 light chain 3 (LC3) is a homolog of yeast Apg8p (or Atg8) which undergoes lipidation to form LC3B from LC3A. The LC3B is known to be crucially involved in the formation of autophagosome and the extent of LC3B is correlated with the extent of autophagosome formation^53^. The curcumin treatment was shown to promote autophagy as evidenced by LC3B formation and its accumulation with E64d assessed by western blotting. Also, there was an increase in LC3B puncta formation after curcumin treatment. mTOR is a negative regulator of autophagy^52^. Curcumin treatment of Ana2014 cells resulted in the decrease in phosphorylation of mTOR which may lead to the activation of autophagy. In our studies, we found that curcumin induced autophagy is ROS dependent manner as pre-treatment with NAC resulted in no LC3B formation and no mTOR phosphorylation.

A large number of studies have been carried towards screening anti-theilerial drugs. Previously, naphthoquinones, namely, parvaquone and menoctone were reported to their anti-theilerial properties^54^. Further, buparvaquone was reported to be effective anti-theilerial drug^55,56^. Buparvaquone induces caspase dependent apoptosis in *Theileria* infected lymphocytes upon parasite death^57^. However, there are reports of resistance to buparavaquone in *Theileria* parasite^58,59^. Our studies suggest that curcumin could be developed as an anti-theilerial drug.

In conclusion, our studies for the first time indicate that curcumin can initiate the process of autophagy and apoptosis in *Theileria* infected bovine leucocytes. We have further shown the mechanism by which curcumin induces autophagy and apoptosis in the *Theileria* infected bovine leucocytes (Fig. 7). Curcumin induces autophagy by inhibiting mTOR phosphorylation in *Theileria* infected bovine leucocytes. Curcumin induces apoptosis through cytochrome c release, activation of caspase 3, caspase 8, and DNA fragmentation in *Theileria* infected bovine leucocytes. Further, antioxidant NAC inhibits both curcumin induced apoptosis and autophagy suggesting the role of ROS. Since curcumin has no effect on uninfected bovine PBMCs, the therapeutic potential of curcumin for curing theileriosis in cattle could further be studied.

## Supplementary information

Figure S1

Figure S2

Figure S3

Figure S4

Figure S5

Table S1

Supplemental Material File #1
