# Supplementary material for: Curcumin induced oxidative stress causes autophagy and apoptosis in bovine leucocytes transformed by Theileria annulata
Source: Cell Death Discov. 2019 Jun 4;5:100. doi: 10.1038/s41420-019-0180-8 (PMC6547749; doi:10.1038/s41420-019-0180-8)
Supplement: Supplementary file 6 — Table S1 [file 41420_2019_180_MOESM6_ESM.docx]

**Table S3: List of primer sequences used in RNA-seq experiment validation**

| S.No | Gene Name | Primer sequence | Annealing temperature (°C) |
| --- | --- | --- | --- |
| 1 | BoSLC1A4 | FP: CTCACCATCGCCATTATCCT  RP: ACTTTGACCTCGCTCAGCTC | 60 |
| 2 | BoTRIB3 | FP: GCTGGAGAACCTGGAAGATG  RP: AGTCCTGGAAGGGGTAGTGG | 60 |
| 3 | BoRAB14 | FP: CCGAAGCTACTACAGAGGCG  RP: TCATACGTCACATCCCGCTG | 50 |
| 4 | BoTXNRD | FP: GCCAAGGAGGCAGCCAAATA  RP: CCTCGACGTTCCACCCATAG | 60 |
| 5 | BoIKBKE | FP: TCTACGGCACCGAGGAATAC  RP: TGCGGTACATGATCTCCTTG | 50 |
| 6 | BoFASN | FP: CTGAGTCGGAGAACCTGGAG  RP: CCATATTGTGTGCCTGCTTG | 60 |
| 7 | BoRAC2 | FP: GTCAGCCCAGCCTCCTATG  RP: GAGTCAATCTCCTTGGCCAGT | 60 |
| 8 | BoMFNG | FP: GAGTGCAAGCTGGGTGGTCA  RP: AATGGAGGGAGTGAAACCTGGA | 60 |
| 9 | BoKPNA4 | FP: TGATGCTGGCAACGAACAGA  RP: GCAGGGAAGTGTGAAAGAGC | 50 |
| 10 | BoGFPT1 | FP: TGAACACAATGAGAGGAAGAGTCA  RP: TGGCAAGCTCCACCATAACA | 60 |
| 11 | BoLCP1 | FP: TGGGCTAACTACCACCTGGA  RP: GCATTCTGCCCTCTGGATGT | 60 |
| 12 | BoMGME1 | FP: TATGTGGGTCTGCTGGACTG  RP: TGTAGGCCACCACGATCAAG | 60 |
| 13 | BoFBH1 | FP: TGACGAGGAGCTGACAATCG  RP: AAGTAGCCATCGTGCGTCAT | 60 |
| 14 | BoTRAF3IP3 | FP: CTATGAGCAGAAGGCCAAGG  RP: GCTCTCCAGCTCTCTGTGCT | 50 |
| 15 | BoCDKL2 | FP: GGTTATCAGTGGGATTGGAT  RP: GAGCTCTATACCATCGTGTT | 50 |
| 16 | BoICAM1 | FP: CTCAGACGCTAAGGTCTAC  RP: TAGACAGTCACGTTCTCTTG | 50 |
| 17 | BoTBP | FP: GCGTTTTGCTGCTGTAATCA  RP: CCCCACCATGTTCTGAATCT | 51 |
| 18 | BoPPIA | FP: TACAGGTCCTGGCATCTTGTCC  RP: CACGTGCTTGCCATCCAACC | 56 |
