# Supplementary material for: Curcumin induced oxidative stress causes autophagy and apoptosis in bovine leucocytes transformed by Theileria annulata
Source: Cell Death Discov. 2019 Jun 4;5:100. doi: 10.1038/s41420-019-0180-8 (PMC6547749; doi:10.1038/s41420-019-0180-8)
Supplement: Supplementary file 7 — Supplemental Material File #1 [file 41420_2019_180_MOESM7_ESM.docx]

**Supplementary figure legends**

**Figure S1. a.** Effect of curcumin on viability of uninfected bovine PBMC, **b.** Curcumin standard absorbance at 428 nm.

**Figure S2. a.** Agarose gel image showing the confirmation of *Theileria* infection in Ana2014 cells through PCR of genomic DNA, **b.** Confirmation of *Theileria annulata* infection in Ana2014 cells by PCR of *Theileria annulata* specific genes TA18945, TA19600 and TA13185.

**Figure S3.** Annexin V-FITC/ PI staining of curcumin treated and untreated cells showing significant increase in apoptotic cell population after curcumin treatment (N=3).

**Figure S4.** Validation of RNA seq experiment by quantitative real time PCR (qRT-PCR). The qRT-PCR analysis of 16 differentially regulated genes was performed. The fold change of the various genes was normalized with endogenous control BoTBP and BoPPIA. a. Increased expression of upregulated genes, b. Decreased expression of downregulated genes. The experiments were performed thrice (biological replicates) and each in triplicates (technical replicates). Data are presented as mean ± SD. The statistical tests were performed with the software Graphpad Prism (Version 7.04). The data were statistically analyzed by multiple t-test. * represents p<0.05, ** represents p<0.01, and *** represents p<0.001, compared with the untreated group.

**Figure S5**. **a.** heat map of differentially expressed genes (using R package, version 3.5.0) of Ana2014 cells with and without curcumin treatment (N=1). Orange-high expression and green-low expression levels.

**Table S1:** List of primer sequences used in RNA-seq experiment validation.
